# Spatiotemporal Variations of Reference Crop Evapotranspiration in Northern Xinjiang, China

**DOI:** 10.1155/2014/931515

**Published:** 2014-08-31

**Authors:** Jian Wang, Xin Lv, Jiang-li Wang, Hai-rong Lin

**Affiliations:** ^1^Agriculture College, Shihezi University, 221 North four Road, Shihezi, Xinjiang 832003, China; ^2^The Key Laboratory of Oasis Ecological Agriculture of Xinjiang Production and Construction Group, Shihezi, Xinjiang 832003, China

## Abstract

To set up a reasonable crop irrigation system in the context of global climate change in Northern Xinjiang, China, reference crop evapotranspiration (ET_0_) was analyzed by means of spatiotemporal variations. The ET_0_ values from 1962 to 2010 were calculated by Penman-Monteith formula, based on meteorological data of 22 meteorological observation stations in the study area. The spatiotemporal variations of ET_0_ were analyzed by Mann-Kendall test, Morlet wavelet analysis, and ArcGIS spatial analysis. The results showed that regional average ET_0_ had a decreasing trend and there was an abrupt change around 1983. The trend of regional average ET_0_ had a primary period about 28 years, in which there were five alternating stages (high-low-high-low-high). From the standpoint of spatial scale, ET_0_ gradually increased from the northeast and southwest toward the middle; the southeast and west had slightly greater variation, with significant regional differences. From April to October, the ET_0_ distribution significantly influenced the distribution characteristic of annual ET_0_. Among them sunshine hours and wind speed were two of principal climate factors affecting ET_0_.

## 1. Introduction

Climate warming is an obvious feature of global climate change [1]. Changes in the distribution of water resource with climate change have a profound impact on human life, production, and ecological environment, which have attracted academic and local government's attention [2, 3].

Reference crop evapotranspiration (ET_0_) is an important indicator which is used to characterize atmospheric evaporation capacity and evaluate drought degree, vegetation water consumption, production potential, water supply, and demand balance [4, 5]. The Food and Agriculture Organization (FAO) of the United Nations defined the reference crop water consumption as the upper evapotranspiration rate of a hypothetical reference crop, supposing that the plant's height was at 0.12 m, resistance of plant leaves was fixed at 70 s/m, and emission rate was 0.23, similar to the evaporation capacity of green grassland with wide surface, that is, consistent height, vigorous growth, complete cover ground, and sufficient moisture [6–8].

Tianshan Mountains, across the center of Xinjiang, divide Xinjiang Uygur autonomous region into south and north parts, while the Northern part is called Northern Xinjiang, characterized by scarce rainfall and dry climate. Shortage of water resource limits sustainable agriculture, animal husbandry, and stable economic development and also changes the local ecological environment. In recent years some studies on spatiotemporal change of ET_0_ in Xinjiang showed up. However, in these papers the emphasis was mostly put on climate and meteorological rate, cumulative departure, Mann-Kendall detection, and geographic information system (GIS) interpolation in a wide range of Xinjiang. And the meticulous research in a limited scope has been very rare [9–11]. In this paper, we calculated 49 years of ET_0_ in Northern Xinjiang through the Penman-Monteith equation, using 1962–2010 daily meteorological data from 22 meteorological stations in the Northern region. Then we used Mann-Kendall test, Morlet wavelet analysis, and ArcGIS spatial analysis function to study the spatiotemporal variation of ET_0_ in Northern Xinjiang. It would provide a foundation for calculating crop water demand and optimizing water-resource allocation, so as to get a reasonable irrigation system in this region.

## 2. Data Source and Analytical Methods

**Figure 1:**
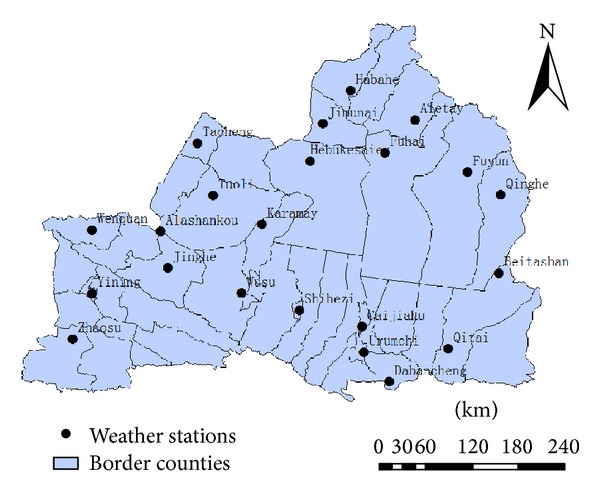
Distribution of meteorological observation stations in Northern Xinjiang.

Northern Xinjiang includes Urumqi, Altay, Tacheng, Changji, Shihezi, Bole, Yili, and other regions, where Junggar Basin locates between Altai, the most Northern part of Xinjiang, and central Tianshan. Average elevation is 770 meters or so. The climate is temperate continental arid or semiarid. There are four distinct seasons, with average annual temperature −4 to 9°C, 150–200 mm annual precipitation, and annual frost-free period 140–185 days. In this area agricultural production is a major industry. This paper analyzed the meteorological data from 22 weather stations in Northern Xinjiang (the locations are shown in Figure 1) from 1960 to 2010, including average daily maximum temperature, average daily minimum temperature, average daily temperature, daily relative humidity, average wind speed, sunshine hours, and station altitude, latitude, and longitude. The Penman-Monteith formula recommended by the Food and Agriculture Organization of the United Nations was used to calculate reference crop evapotranspiration for Northern Xinjiang:

(1) $$\begin{array}{c} \text{E}{\text{T}}_{0}=\frac{0.408\Delta \left(R_{n}-G\right)+\gamma \left(900/\left(T+273\right)\right)U_{2}\left(e_{s}-e_{a}\right)}{\Delta +\gamma \left(1+0.34U_{2}\right)}, \end{array}$$

where ET_0_ is reference crop evapotranspiration (mm day^−1^); Δ is slope vapor pressure curve (kPa °C^−1^); *R*_*n*_ is net radiation at the crop surface (MJ m^−2^ day^−1^); *G* is soil heat flux density (MJ m^−2^ day^−1^); *γ* is the psychrometric constant (kPa °C^−1^); *T* is mean daily air temperature at 2 m height (°C); *U*_2_ is wind speed at 2 m height (m s^−1^); *e*_*s*_ is saturation vapor pressure (kPa); and *e*_*a*_ is actual vapor pressure (kPa).

Mann-Kendall [12] test is a nonparametric test method, in which samples do not need to follow a certain distribution patter and will not be affected by the interference of a few of outliers. So it is very suitable for analyzing the nonnormal distribution. It has been wildly used for its wide detection range and high degree of quantitative and convenient calculation meanwhile it can identify when and where the mutation appears while the mutation analysis is carried out. Statistics of UF_m_ algorithm was shown in the reference crop evapotranspiration (ET_0_). In this paper, given significance level *α* equal to 0.05, after checking the table of normal distribution we could find that the distribution range of value U_0.05_ was equal to 1.96. If the absolute value of UF_m_ was bigger than U_0.05_, it indicated that there was a clear trend change sequence. All UF_m_ values were shown in a curve UF. This test sets up two series, a progressive one (UB) and a backward one (UF). The statistic curve, UF, UB, and two straight lines, plus or minus 1.96, were drawn in the same graph. When they exceeded the critical lines, it meant there was a significant upward or downward trend happening. For UF, if the value was greater than 0 it indicated that UF series were rising, conversely downward trend. If the two curves of UF and UB intersected between the critical lines, then the moment of the intersection might be the mutation start time. In this paper Mann-Kendall test was used to analyze mutation and trend of ET_0_ sequence.

Wavelet analysis [13] was developed over nearly 28 years and is a type of signal frequency with localization analysis, used to ascertain local characteristics of periodic change. One can clearly discern a cycle of temporal change by it. So wavelet analysis is very suitable for analyzing multiple time scales. Complex wavelet coefficients more realistically reflect the scale distribution of periodic size and cycles in the time domain, and the Morlet wavelet transform has seen years of development, so they have become turn into mature analysis tools. In this paper the Morlet wavelet was used to study the characteristics of ET_0_ sequence scale and periodicity, with wavelet generating function *ϕ*(*t*) = *e* ^*iω*_0_*t*^ *e* ^−*t*^2^12^. Here, *ω*_0_ is a constant, *i* is the imaginary part units, and *t* is time.

Kriging space interpolation (kriging) is an ArcGIS software geostatistical interpolation method, which has unique advantages in statistics ideological space over the local interpolation method and spatial analysis randomness and structural variables [14]. The essence of the original data and structural characteristics of the variogram is regionalized variables, not the value of sampling-point regionalized variables from linear unbiased optimal estimation [15]. Given the estimated value of any one space variable point *Z*_*x*_^*i*^ and *N* within its sphere of influence, the effective observation values *Z*(*X*_*i*_) are determined by their respective weights:

(2) $$\begin{array}{c} Z_{x}^{*}=\overset{n}{\underset{i=1}{\sum }}\lambda_{i}Z\left(X_{i}\right), \end{array}$$

where *λ*_*i*_ is the meteorological observation and *Z*(*X*_*i*_) is the weight coefficient. The weight coefficients are obtained by ordinary or simple kriging equations. The weight coefficient is determined by the spatial structure of variables. And the spatial structure of variables is expressed in half variant function *γ*(*h*), that is,

(3) $$\begin{array}{c} \gamma \left(h\right)=\frac{1}{2N\left(H\right)}\overset{N(h)}{\underset{i=1}{\sum }}{\left(Z\left(X_{i}\right)-Z\left(X_{i}+h\right)\right)}^{2}, \end{array}$$

where *H* is a distance vector and *N*(*h*) is the number of data points separated by distance *h*.

## 3. Results

### 3.1. Interannual Variability

**Figure 2:**
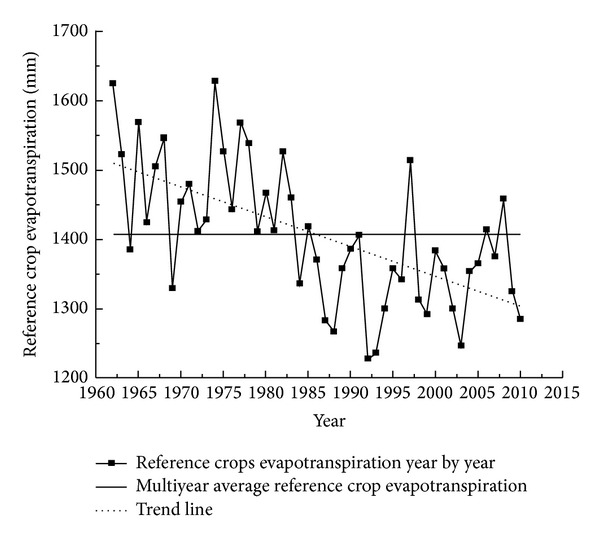
Annual variation of average ET_0_ in Northern Xinjiang.

We obtained annual average ET_0_ of all studied weather stations over 49 years (1962–2010).  Figure 2 shows interannual variation curve and linear trend of average ET_0_ in the study area. The annual average ET_0_ was 1407.4 mm with fluctuation range from 1228.4 to 1628.7 mm. The minimum was about 1228.4 mm turning up in 1992 while the maximum was about 1628.7 mm in 1974. The ratio of maximum to minimum was 1.33. The linear trend line indicated that ET_0_ went downward over time with a decrease extent about 1.3 mm/a. The Mann-Kendall test was performed on the ET_0_ sequence. The result showed that statistic *Z* was equal to −4.32 and less than −1.96, indicating a significant downward trend (confidence level 95%).  Table 1 showed the Mann-Kendall test result of ET_0_ sequence in a confidence level of 95%. ET_0_ values of stations in Fuyun, Beitashan, and Tacheng showed a significant increasing trend while ET_0_ in other sites showed a decreasing trend. According to the above the study area was divided into two regions, that is, ET_0_ decreasing area and increasing area.

### 3.2. Characterization of Mutations

**Figure 3:**
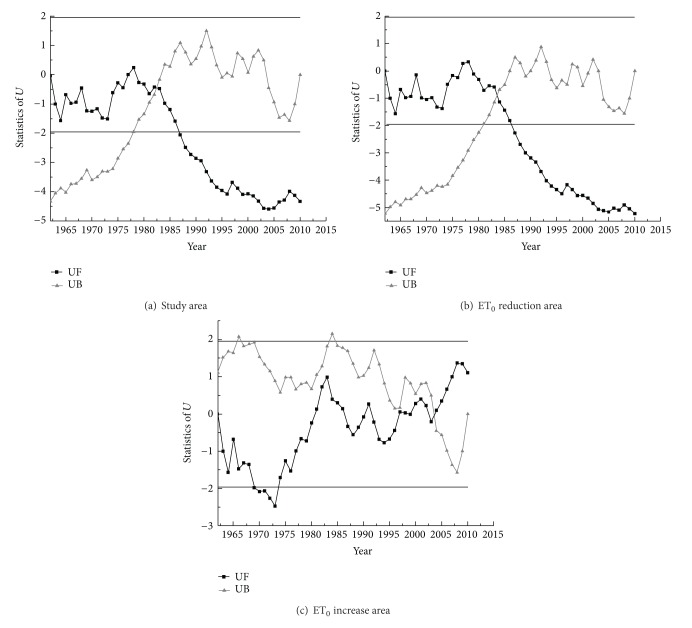
Mann-Kendall test of average ET_0_.

In order to estimate the significant downtrend of reference crop evapotranspiration in the study area and the relationship between ET_0_ decrease or increase and mutation, mutation analysis of ET_0_ sequence was carried out by means of the Mann-Kendall test (Figure 3).  Figure 3(a) showed that the average ET_0_ in the study area began to decline in 1979, and it dropped significantly in 1987. The two curves of UF and UB intersected at about 1983. The average ET_0_ mutation of the study area showed up in this year, indicating a sudden ET_0_ decline in the early 1980s. The sudden decrease was about 128.6 mm.  Figure 3(b) showed that the regional-average ET_0_ began to decline in 1979, with a significant reduction beginning in 1987. The mutation point appeared around 1984, with a decrease approximately 160.7 mm.  Figure 3(c) showed that the mutation time of average ET_0_ showed up in 2004 in the ET_0_ increasing area and began to rise from 1981 with a sharp rise in 2008 and the increase was about 153.4 mm.

Comprehensive analysis of Figures 3(a), 3(b), and 3(c) showed that the mutation time of the reference crop evapotranspiration in ET_0_ reduced area was relatively similar to the whole study area, while that in ET_0_ increased area was totally different from the whole study area.

### 3.3. Cycle Characteristics

**Figure 4:**
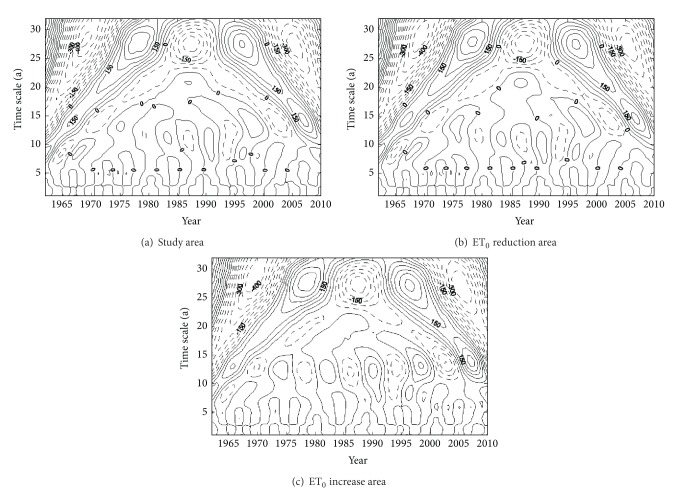
Morlet wavelet transform of average ET_0_.

Figures 4(a), 4(b), and 4(c), respectively, represented an average ET_0_ Morlet wavelet transform coefficient (real part) contour map of the study area, ET_0_ decrease area, and increase area. The real part of the wavelet reflected the time-scale signal characteristic at varying times for the strength and phase information. The positive phase represented the period with ET_0_ more than normal, the negative phase represented the period with ET_0_ less than normal, and zero wavelet coefficients corresponded to the mutation point.  Figure 4(a) showed that at different time scales, ET_0_ sequence change had different characteristics and the phase structure had a strong cycle around 28 years. On this time scale, there were some increases during 1962–1966, 1977–1997, and 2008–2010. There were decreases during 1967–1976 and 1967–2007, that is, the negative phase. So there was a high-low-high-low-high cycle in the process. Mutation points turned up in 1965 and 2002. Figures 4(b) and 4(c) showed that at different time scales the change characteristics of average ET_0_ in the increase area and decrease area were similar to those in the study area. The results of primary period analysis of ET_0_ in the study area, increase area, and decrease area during 1962–2010 were shown in Table 2. The results showed that both primary period and secondary period of ET_0_ in the reduction area were the same as those in the study area, while only primary period of ET_0_ in the increase area was the same as the study area and secondary period was very close to the study area.

### 3.4. Spatial Variation

**Figure 5:**
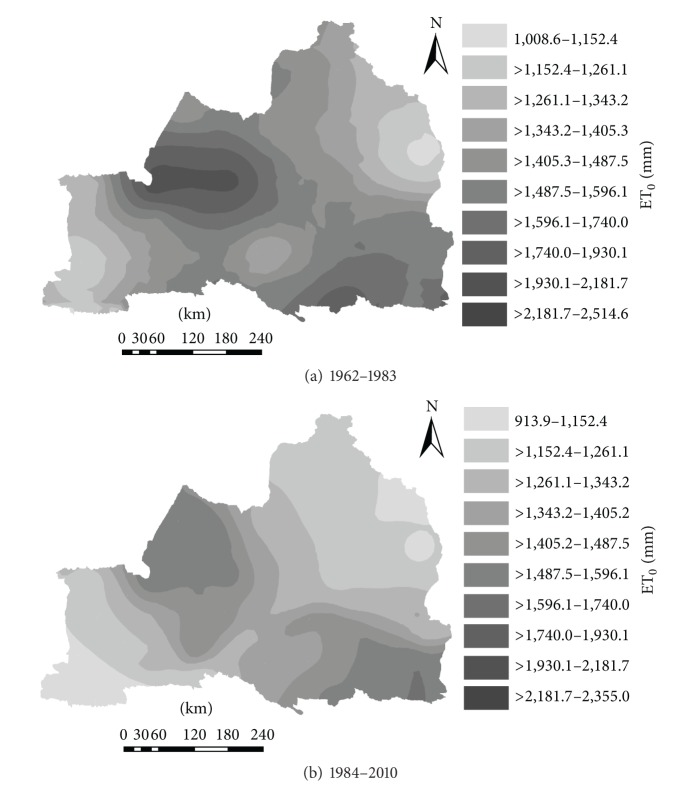
Spatial distribution of average annual ET_0_ between 1962 and 1983 and 1984 and 2010 in Northern Xinjiang.

Using the spatial kriging interpolation method of ArcGIS 9.3 based on ET_0_ spatial rasterization, we obtained ET_0_ values on each grid and also ET_0_ continuous spatial distribution. It provided detailed data for the ET_0_ spatial variation analysis in the Northern agricultural area of Tianshan Mountains, Xinjiang. To analyze the spatial distribution characteristics of mean ET_0_ in many years before and after mutations in the study area, we used Arc GIS 9.3 to map the characteristics by using spatial interpolation (Figure 5).  Figure 5(a) showed that before the mutation (1962–1983) spatial distribution characteristics of ET_0_ were that the value of ET_0_ was low in the northeast and southwest, but it was high in the southeast and west. Meanwhile, from the northeast and southwest to the middle ET_0_ slowly increased and the average variation ranges of ET_0_ over many years were 1008.6–2514.6 mm; the high value area in the southeast and west was within 1596.1–2514.6 mm; and the low value area in the northeast and southwest was within 1008.6–1405.3 mm. We could see that there were significant regional differences in the study area.  Figure 5(b) showed that after mutation (1984–2010) ET_0_ spatial distribution characteristics were that the ET_0_ in the northeast and southwest was low and the ET_0_ significantly declined compared to it before mutation. The average ET_0_ for many years was in the range of 913.9–2355 mm, with the low value area of the southwest and northeast from 913.9–1191.3 mm and the high value area within 1317.5–1621.8 mm. Therefore, there was a significant difference.

Overall, spatial distribution of ET_0_ had a significant change before and after mutation. Reduction areas were as follows. Ili Valley in southwest declined by 92.8–124.8 mm. Altay region in the northeastern Xinjiang declined by 20.8–261.4 mm. Along the north slope area of Tianshan Mountains declined by 86.6–379.5 mm. And the Western declined by 156.6–24.15 mm.

**Figure 6:**
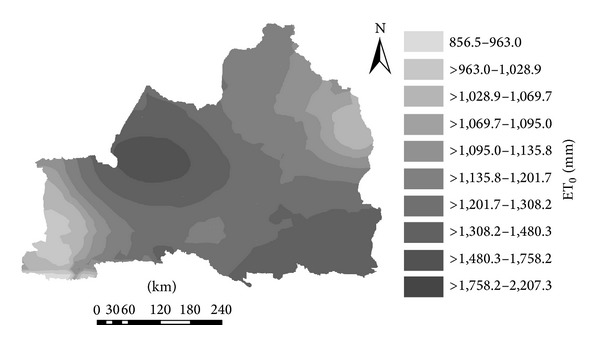
Spatial distribution of average monthly ET_0_ from April to October of 1962–2010 in Northern Xinjiang.

The ET_0_ from April to October accounted for more than 80% of the annual ET_0_ of the main seasonal crop in Northern Xinjiang. Thus, the analysis of the ET_0_ spatial distribution in these months was of great significance. Using Penman-Monteith equation and meteorological data in combination with the monthly-average ET_0_ and the sum ET_0_ from April to October of the study area, the ET_0_ spatial distribution for April to October was produced with ArcGIS 9.3 (Figure 6). Figure 6 showed that the spatial distribution characteristics of ET_0_: ET_0_ in Midwest and southeast flanks were high, in southwest and northeast were low, and in Midwest and East to the middle were decreasing. ET_0_ changed in the range of 856.5–2207.3 mm, while Midwest and southeast flanks, the high value area, changed in the range of 1201.7–2207.3 mm, northeast and southwest, the low value area, changed in the range of 856.5–1095 mm. It showed distinct regional differences.

### 3.5. Spatiotemporal Variation Causes

**Figure 7:**
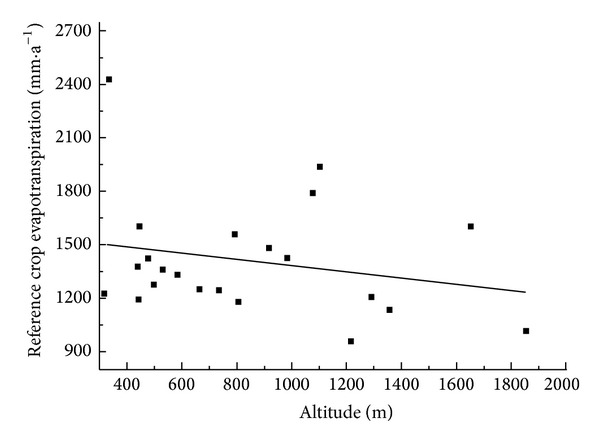
Tendency of ET_0_ with altitude.

ET_0_ spatiotemporal change was affected by a variety of factors. Six important meteorological and topographical factors (altitude) of them were selected in this paper. Factor analysis results were shown in Tables 3 and 4 and Figure 7.  Table 3 showed that wind speed and sunshine hours had significant positive correlation with ET_0_ in the study area. Relative humidity and rainfall had highly significant negative correlations with ET_0_. And wind speed and ET_0_ in the reduction area showed a significant positive correlation, while relative humidity and rainfall had highly significant negative correlations with ET_0_. In other words, ET_0_ increase, wind speed, sunshine hours, and ET_0_ had highly significant positive correlations, but there was a negative correlation between relative humidity and ET_0_. According to Table 3, the partial correlation between meteorological factors and ET_0_, and Table 4, the main meteorological factor trend with time, results showed that ET_0_ decreased mainly because of a significant decline of wind speed. Within the context of the overall ET_0_ decline, ET_0_ values of Tacheng, Fuyun, and Beitashan with special upward trends were due to wind speed and there was no significant decrease of sunshine hours.

From Figure 7, ET_0_ decreased with altitude but changed little in space. The linear trend line equation was *y* = −0.1743*x* + 1554.3, with nonsignificant correlation coefficient −0.229. In Northern Xinjiang, elevation was not the main cause of significant regional differences of ET_0_.

## 4. Discussion

Many studies on ET_0_ in the northwest of China showed that ET_0_ of Northern Xinjiang was usually around 1800 mm [16, 17]. In this study, the maximum value of ET_0_ in Dabancheng and Alashankou reached 1935.8 mm and 2426.9 mm differently. As far as the reasons were concerned, firstly, previous studies had generally limited number meteorological of stations selected (roughly 13–17 sites) and limited coverage [16, 17]; secondly, “*Xinjiang Meteorological Handbook*” compiled by Zhang showed that annual average wind speed of Dabancheng was 4-5 m/s, annual average wind speed of Alashankou was up to 6 m/s, and “Baili wind zone” in Eastern of Xinjiang had an average annual wind speed at 5–8 m/s [18]. The wind speed was one of the main meteorological factors affecting ET_0_ and there was a significant positive correlation between them. Therefore, ET_0_ values of those areas were high. Zhang and Pu had found that the ET_0_ of wind line, outlet and the Gobi desert zone in “Baili wind zone” of Eastern Xinjiang was up to 1800–2600 mm, where the ET_0_ was the largest in Xinjiang. It was similar to the conclusions of this study [11].

The multitemporal and spatial variation analysis of ET_0_ revealed that wind speed and sunshine hours were the main influences on ET_0_ in Northern Xinjiang [19–22]. Maximum and minimum temperature effects were relatively weak and consistent with earlier studies [23–25]. Relative humidity and rainfall had a negative correlation with ET_0_. This might attribute to that precipitation had increased, relative humidity had gotten high, and climate was becoming warmer and wetter during recent years in Northern Xinjiang [26]. Maximum temperature affected ET_0_ somewhat, but not significantly in Northern Xinjiang in contrast to the traditional perception [13, 27, 28]. This is the “evaporation paradox” phenomenon [29]. Wind had a significant impact on ET_0_ in windy areas. Therefore, farmland shelterbelts and other measures were important to agricultural production in such areas.

Some scholars hold that elevation had a significant influence [20], but this was not significant in the study area. This finding might be due to the complex terrain of Northern Tianshan Mountains, Yili Valley and Junggar basin, Gurbantünggüt Desert, and Altai. Additional research needs to be done to confirm this idea.

Several studies have shown that the development of irrigation in arid zone oasis agriculture has resulted in a reduction in wind speed and an increase in relative humidity. As a consequence of these changes, reference crop evapotranspiration in this region has decreased [30]. While different irrigation systems had different operating modes, irrigation and drainage system could have an influence on the soil moisture in field, farmland microclimate, and crops' demand for water. In the conventional broad irrigation evapotranspiration was more than spray irrigation, drip irrigation, and other water-saving irrigation modes. Therefore, irrigation modes would have some impacts on the reference crop evapotranspiration [31].

The agriculture in Northern Xinjiang belongs to irrigated agriculture. In the early 1950s the irrigation area of Xinjiang was only 0.6 million hm^2^ [32]. From the beginning of the 1980s, with the growing investment in water conservancy facilities and construction, irrigation area gradually expanded. In the beginning of 1990s drip irrigation technology was introduced in Northern Xinjiang; the drip irrigation was proven to be the best water-saving irrigation technique in Northern Xinjiang, which has been successfully applied to a variety of crops, such as cotton, corn, tomato, and wheat at present [33]. According to statistics, the amount of water diversion in Northern Xinjiang reached 11.71 billion m^3^ and the irrigation area peaked up to 1.53 million hm^2^ in 2009 [34], but it only accounts for about 40% of the irrigated area [34].

In order to further improve the accumulated temperature and reduce evaporation in production, spring sowing of thermophilic crops (cotton, corn, processed tomatoes, etc.) used drip irrigation under film. Compared to the irrigation mode with ditches in the past, this irrigation mode not only reduced the water loss in conveying and water surface evaporation during flood irrigation. Moreover in this mode high irrigation frequency and small amount of irrigation every time made relatively less change in soil moisture before and after irrigation, that is, soil evaporation was almost unchanged; so the dominant factor affecting the crop evapotranspiration was just crop transpiration [35]. Meanwhile, covering film also reduced the surface evaporation in field. Because it formed a different field microclimate, which could have an influence on reference crop evapotranspiration in some extent.

As the drip irrigation mode has great popularization potential, whether it will contribute to the reference crop evapotranspiration change and to what extent in the future is still unknown and needs further exploration and deeper research.

In the recent 49 years in Northern Xinjiang ET_0_ overall showed a downward trend, and annual changes of ET_0_ in the world and most parts of China were consistent [9–11, 23, 36–39]. Thus, if the crop coefficient is unchanged, it will make the annual water need of main crops, such as cotton, wheat, corn, and oil sunflower, decrease. Although this can ease the pressure on agricultural water use in certain extent, but the agriculture in the study area belongs to irrigated agriculture. So Northern Xinjiang should set up a relative optimizing irrigation system based on the different local situation under the context of global climate change.

According to Mann-kendall test results there would be two kinds of ET_0_ change trends with time in Northern Xinjiang. One was increase where three sites were far away from each other, and the other was reducing surrounding the increase area. This phenomenon needs to be further investigated in the future.

## 5. Conclusions

1. Time-varying characteristics: The multiyear average ET_0_ was 1407.4 mm in the study area, with a range of 1228.4–1628.7 mm. The results of Mann-Kendall test showed ET_0_ values of 19 weather stations that had a significant downward trend with time while the other three stations had an upward trend. The mutations of average ET_0_ in the study area, reduction area, and increase area appeared, respectively, in 1983, 1984, and 2004. Morlet wavelet analysis showed a corresponding phase structure, with a strong cycle around 28 years. On this time scale, there were increases between 1962 and 1966, 1977and 1997, and 2008 and 2010. Meanwhile there was a decline between 1967 and1976 and 1967 and 2007, that is, the negative phase. There were high-low-high-low-high cycles in the process. The principal ET_0_ cycle was 28a, and the minor cycle was around 15a. The ET_0_ of the reduction area had the same main period and hypoperiod as the study area, and the increase area only had a similar hypoperiod to the study area.
2. The results of spatial variation characteristics showed that compared to the spatial distribution of average ET_0_ before and after mutation, the average ET_0_ values in northeast and southwest of Northern Xinjiang were low while the average ET_0_ of southeast part was high. Before mutations the average ET0 in the northeast and southwest of Xinjiang north to the middle gradually increased. The average ET_0_ spatial distribution from April to October was consistent with the distribution of annual ET_0_.
3. In general, the main meteorological factors impacting ET_0_ were sunshine duration and wind speed. The ET_0_ values of Tacheng, Fuyun, and Beitashan stations showed significant increasing trend because of nonsignificant declines in sunshine hours and wind speed. Wind speed significantly affected ET_0_; so the shelterbelts and other measures to reduce wind speed are very important for agricultural production in Northern Xinjiang and Tianshan regional farmland. With the continuous development of 3S (GIS, RS, and GPS) technologies, their application in the agriculture will become more common and sophisticated. This would give increasingly accurate spatiotemporal variation results of ET_0_, including those places with complex terrain.

## Figures and Tables

**Table 1:** Mann-Kendall test of ET_0_ at each meteorological observation station.

Weather stations	*Z*-value statistics	Weather stations	*Z*-value statistics
Alashankou	−3.71	Wenquan	−4.52
Ürümqi	−2.98	Karamay	−5.78
Qinghe	−1.95	Altay	−4.02
Hebukesai'er	−4.83	Fuhai	−4.14
Yining	−3.07	Shihezi	−1.83
Qitai	−5.43	Jimunai	−3.78
Dabancheng	−3.21	Habahe	−5.05
Zhaosu	−3.05	Tuoli	−2.26
Caijiahu	−3.29	Fuyun	1.40
Wusu	−5.62	Beitashan	0.61
Jinghe	−4.38	Tacheng	0.33

**Table 2:** Period statistics.

	Primary period/a	Secondary period/a
Study area	28	15
ET_0_ reduction area	28	15
ET_0_ increase area	28	14

**Table 3:** Partial correlation coefficient between ET_0_ and meteorological factors.

	Wind speeds	Relative humidity	Sunshine duration	Highest temperature	Lowest temperature	Rainfall
Study area	0.65∗∗	−0.68∗∗	0.23∗	0.19	−0.04	−0.23∗
ET_0_ reduce area	0.76∗∗	−0.62∗∗	0.07	0.13	0.03	−0.32∗
ET_0_ increase area	0.50∗∗	−0.70∗∗	0.25∗	0.11	0.09	−0.15

*indicates significant (alpha = 0.05). ∗∗denotes very significant (alpha = 0.01).

**Table 4:** Mann-Kendall test of meteorological factors significantly associated with ET_0_.

	Wind speeds	Relative humidity	Sunshine duration	Rainfall
Study area	−8.01	0.40	−3.57	5.14
ET_0_ reduce area	−7.71	0.59	—	5.05
ET_0_ increase area	−2.53	−0.67	−3.08	—
